# Physical activity, fitness, sedentary behaviour, and brain health: a systematic review of Mendelian Randomisation studies

**DOI:** 10.1038/s44437-026-00013-2

**Published:** 2026-11-10

**Authors:** Adrià Túnez, Margot P. van de Weijer, Joseph Firth, Karin J. H. Verweij, Jorien L. Treur

**Affiliations:** 1 Department of Psychiatry, Amsterdam UMC location University of Amsterdam, Amsterdam, The Netherlands; 2 Amsterdam Public Health Research Institute, Amsterdam, The Netherlands; 3 Amsterdam Neuroscience Research Institute, Amsterdam, The Netherlands; 4 Division of Psychology and Mental Health, Faculty of Biology, Medicine & Health, University of Manchester, Manchester, UK; 5 Greater Manchester Mental Health NHS Foundation Trust, Manchester Academic Health Science Centre, Manchester, UK

**Keywords:** Diseases, Neurology, Neuroscience

## Abstract

While observational and interventional research has linked physical activity (PA) to better psychological and neurocognitive outcomes (termed ‘brain health’), the causal impact of day-to-day PA on brain health remains unclear. We systematically reviewed Mendelian Randomisation (MR) studies testing causal effects of PA, sedentary behaviour and physical fitness on brain health outcomes. Searches in PubMed and medRxiv identified 1114 articles. Sixty-one met the inclusion criteria, with low (52%) to moderate (46%) study quality scores. Thirty studies found only null or directionally opposite effects of PA on brain health outcomes, although with promising evidence emerging for aerobic exercise reducing depression risk (3/3 studies), physical fitness improving mental health (6/8 studies) and cognitive (9/12 studies) outcomes. Results were partially influenced by pleiotropy inherent to complex phenotypes. Overall, the MR evidence for causal effects of PA on brain health remains limited and mixed, highlighting the need for improved phenotyping and triangulation with complementary causal designs.

## Introduction

The term ‘brain health’ is defined as an umbrella term encompassing all psychiatric, mental well-being and neurocognitive outcomes^1–3^. Mental health disorders are one component of brain health. They affect almost 30% of individuals across the lifespan^4^ and account for 32% of all years lived with disability and 13% of disability-adjusted life years worldwide^5^. The prevalence of the most common mental health disorders (i.e. depression and anxiety) has been increasing in the last 20 years in the United States and Europe^6,7^. Often, individuals with a mental health disorder also exhibit cognitive dysfunction^8,9^ which can lead to loss of independence, affecting their ability to live a fulfilling life. While treatment and detection are constantly improving, many individuals do not achieve remission with standard treatment ^9^.

Alongside the development of novel pharmaceutical and therapeutic treatments, the field of psychiatry has placed more focus on daily life activities that influence health, including physical activity (PA)^10,11^. The World Health Organization (WHO) defines PA as any bodily movement produced by skeletal muscles that requires energy expenditure, consistent with foundational definitions in the field^12,13^. A subset of PA is exercise, which refers to structured activities aimed at improving or maintaining physical fitness, while physical fitness itself reflects a set of attributes such as cardiorespiratory capacity and muscular strength^13^. In addition, sedentary behaviour is defined as any waking behaviour characterised by low energy expenditure in a sitting or reclining posture, and is conceptually independent from physical inactivity, which refers to not meeting recommended levels of PA^14^.

According to cross-sectional scientific evidence accrued over the past century, higher levels of PA—particularly in the form of leisure-time or structured exercise—are positively associated with psychological and physical well-being^15,16^. Although robust causal evidence is limited, guidelines around PA have already been adopted by several institutions. For instance, both the US Physical Activity Guidelines for Americans^17^ and the UK Chief Medical Officers’ Physical Activity Guidelines^18^ recommend attaining at least 150 min of moderate-to-vigorous intensity PA per week for reducing the risk of depression. Similarly, the WHO currently recommends regular exercise as a means to maintain a healthy cognitive state^19^.

The available evidence for causality comes from randomised intervention studies, which show that exercise programs benefit various outcomes, including cognitive functioning, depression (severity), sleep quality and psychiatric symptoms^20–22^. Interventions typically include aerobic exercise, resistance training and mixed-modality programs, delivered in both clinical and community-based populations, with frequencies ranging from 1 to 5 sessions per week and durations spanning 3 to 16 weeks^23^. Meta-analytic evidence indicates that PA is associated with moderate reductions in depression and small reductions in anxiety across non-clinical populations^24^. Similarly, a large meta-analysis of RCTs across neurological and psychiatric conditions reported significant improvements in depressive symptoms and small but consistent benefits for cognitive domains^25^. Similar results have been found across different age groups and settings^26^. However, some intervention designs have been identified as low in quality^27^, with limitations such as selection bias, attrition bias and inconsistent results in long-term interventions^28^. In addition, it is not certain yet whether daily PA, not manipulated through an intervention, also causally influences mental health or whether people in a healthier mental state feel more inclined or motivated to engage in higher levels of PA.

An additional source of result heterogeneity lies in how PA and related constructs are measured. For instance, self-reported measures can differ greatly from device-based measurements (e.g. reporting exercise times per week vs recorded exercise as obtained from an accelerometer). In a meta-analysis on self-reported PA and mental health, leisure-time and school-based PA were positively associated with better mental health, while work or household-related PA were either negatively associated or showed no association with mental health^29^. In a large-scale cohort study, different patterns of associations between self-reported and device-measured PA and mental health were reported^30^. In summary, many questions remain on the extent to which effects of PA and related constructs vary across the type and setting^31^.

In the past few years, genetically informed research has gained popularity in PA research, providing valuable alternatives to RCTs. A Genome Wide Association Study (GWAS) is used to identify specific genetic variants that are associated with risk for a disease, or any other human trait, with the goal of better understanding its biological underpinnings^32^. Multiple GWASs of PA-related constructs have been published, including device-measured PA, self-reported exercise, handgrip strength, sedentary behaviour and others. The resulting *summary data* of a GWAS (i.e. the effect estimates summarised across all individuals for all analysed genetic variants) can be used for follow-up analyses, including a causal inference method: Mendelian Randomisation (MR). MR uses the random nature of genetic inheritance to study causality in observational epidemiological studies^33^. When certain underlying assumptions hold, confounding and reverse causality are circumvented. MR has been used to assess causal relationships with measures of brain health in multiple studies. However, an overview of the outcomes of such studies and consistency across different measures of PA and related constructs is currently lacking. Therefore, in this study, we review all available MR literature on the effect of PA, fitness and sedentary behaviours, on different ‘brain health’ outcomes, in order to answer the following questions: (1) Do MR studies find evidence for causal effects of PA on brain health? (2) Are there any differences in results between objectively-measured vs. self-reported PA?

## Results

### Searches

**Fig. 1:**
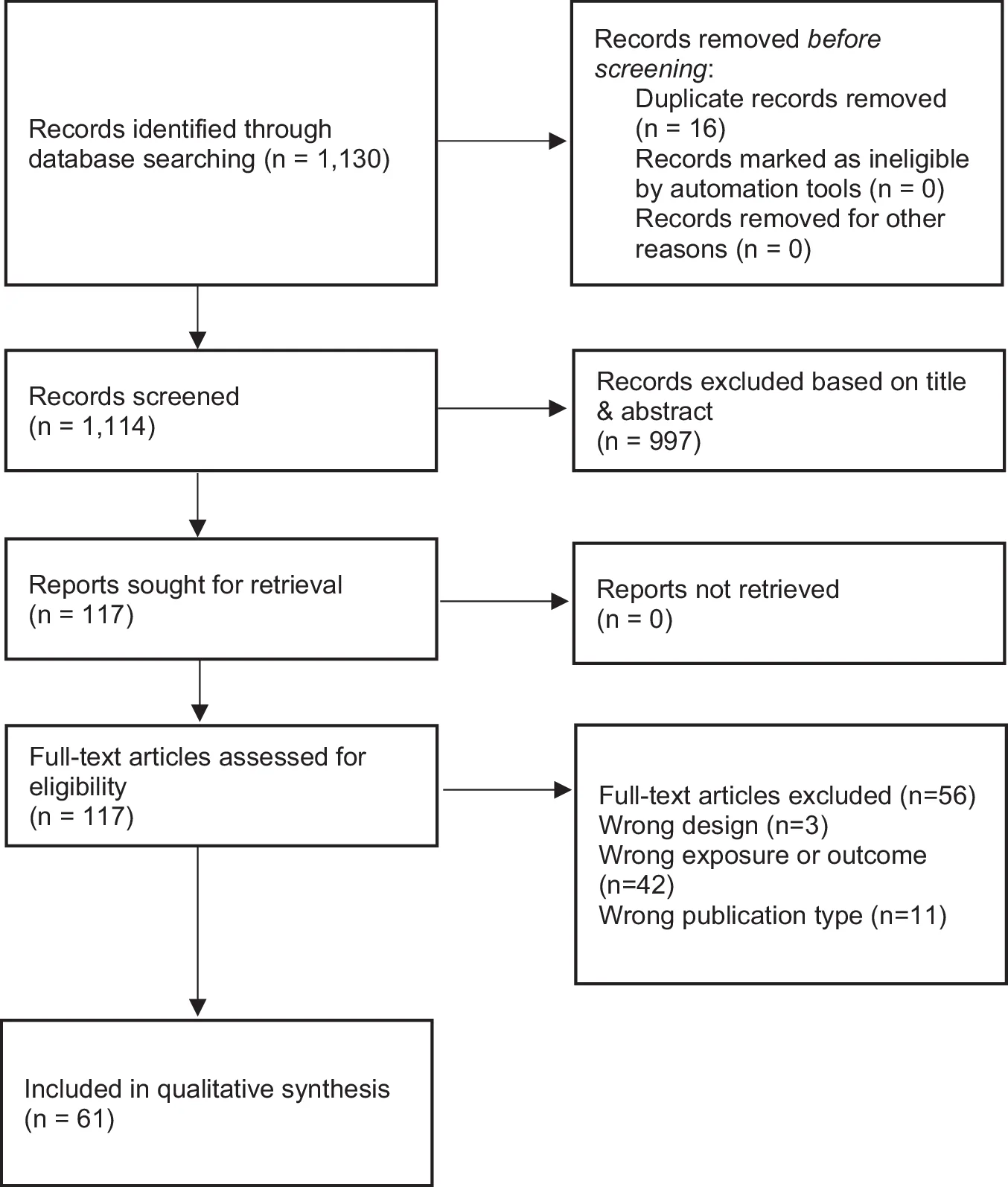
PRISMA flow diagram. PRISMA flow chart demonstrating the selection of articles included for qualitative synthesis.

A total of 1130 articles met our search query, of which 1114 were unique (Fig. 1). Of these, 997 were excluded based on title and abstract, mainly due to wrong exposures and outcomes or wrong design (e.g. review or no MR). The remaining 117 articles were retrieved and assessed for eligibility. During screening of the full text, another 56 studies were excluded. Of the final 61 studies included in qualitative synthesis, 27 investigated cognitive outcomes, 30 investigated psychiatric outcomes and 4 investigated both (see Supplementary Table S1).

### Descriptives

Out of the 61 studies, 32 (52%) received a final score of insufficient (–), 28 a sufficient (±) score and 1 a good (+) score (Supplementary Table S2). While the sample sizes were considered large in 76% of the studies, very few cases (11%) had a sufficiently strong instrument strength (based on a combination of *p*-value threshold, number of SNPs, biologically informed, F statistics and variance explained reported and sufficient). Mostly due to either relaxed *p*-value thresholds, a small number of SNPs or a lack of reported statistics. Only 14 studies presented extensive sensitivity analyses.

Given the limited availability of well-powered PA-related GWASs and brain health measures, 35 studies used the same or partly the same data sets to obtain genetic estimates for both exposures and outcomes. Many of the remaining studies used the same datasets for either the exposure or outcome. In fact, all studies included at least one trait obtained from a GWAS from UK Biobank data, albeit not necessarily from the exact same sample of people. This means that the causal findings presented should not be regarded as (completely) independent (overlap is indicated in Supplementary Table S3).

**Fig. 2:**
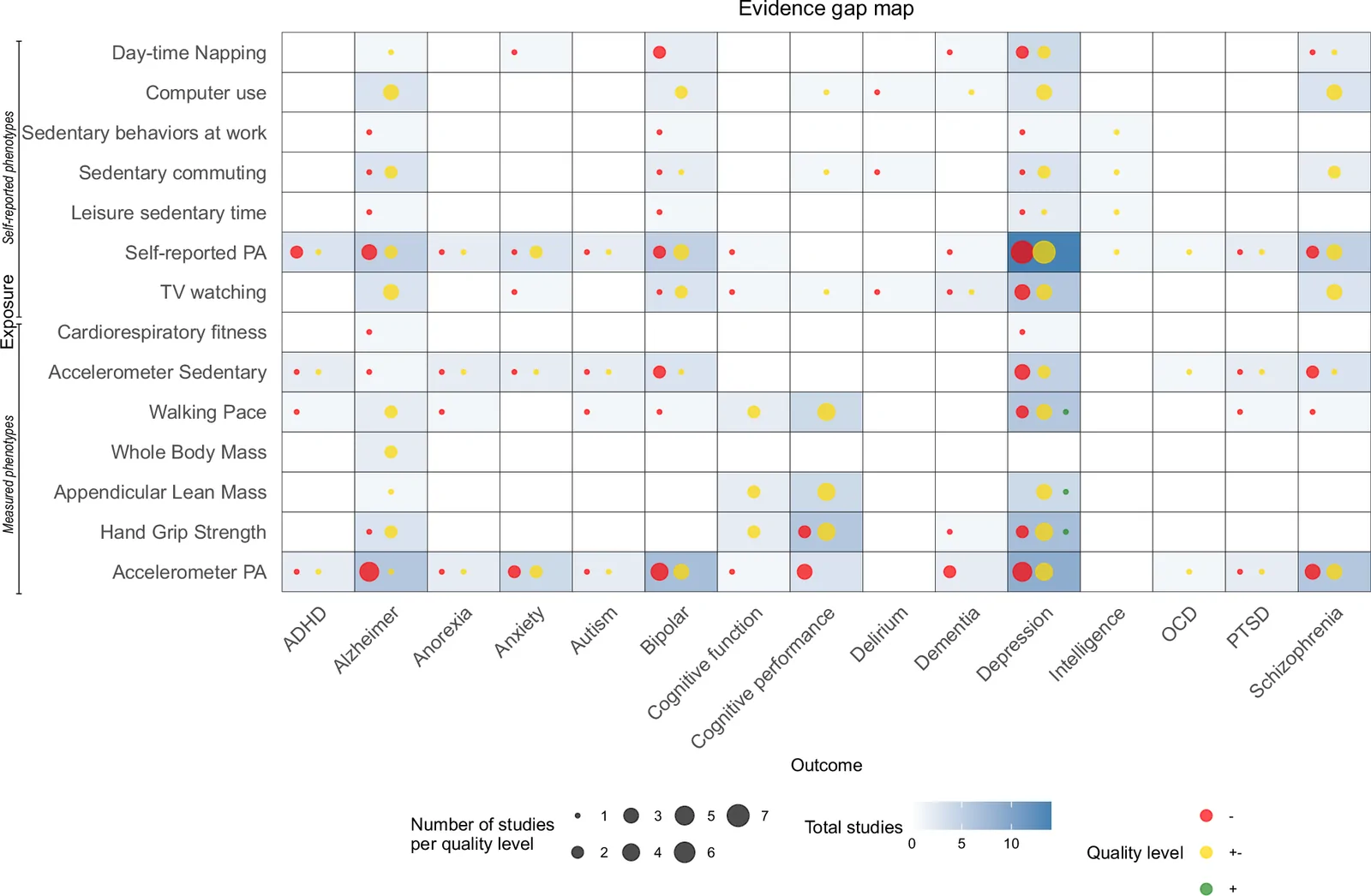
Evidence gap map representing the number of studies (in total and per quality level) for each exposure and outcome of interest. Larger bubbles indicate a larger number of studies, while colour coding reflects study quality (green representing higher-quality and red lower quality studies). Figure created with Adobe Illustrator.

An overview of the studied exposure—outcome pairs can be seen in the evidence gap map (Fig. 2). Depression, schizophrenia, AD, cognitive performance and bipolar disorder were the most studied brain health outcomes, while self-reported PA and accelerometer-measured PA were the most studied exposures. The overall quality of MR studies seems to be similar between self-reported and device-measured phenotypes. This is mostly due to the larger sample size and number of SNPs of self-reported phenotypes, and the better phenotype measurement in device-measured phenotypes.

### Effects of device-measured and self-reported PA

There were mixed effects of accelerometer-measured PA on depression. While most studies^34–38^ reported weak evidence that PA decreases depression risk, some studies reported no effects^39,40^. The quality of the studies reporting evidence for causality was, on average, rated lower than those that did not (− vs. ±), with results mostly being inconsistent across sensitivity methods. Additionally, Choi et al.^34^ and Iob et al.^36^ evaluated the same exposure/outcome summary statistics. Casanova et al.^35^ and Ba et al.^37^ did too. No evidence for the effect of PA on schizophrenia^41–44^ or bipolar disorder was reported^36,40^. One study reported measured PA decreasing risk of bipolar disorder^45^, but its quality was low (−). No effects were found on anxiety or PTSD^36,40^.

There were also mixed effects of self-reported PA on depression risk. While some studies found evidence for protective effects^46–50^ most found no effects^36,37,39,48,51–54^. When comparing different intensities, the most consistent finding was on the effects of aerobic exercises^48,49,55^, while no effect was found from other strenuous or lower intensity exercises on depression^50^. In one study^56^, heavy DIY activities were found to be associated with a lower risk of bipolar disorder. Studies reporting no effect were, on average, similar in quality to those that did, while the results of those that found effects were consistent across sensitivity methods. Self-reported PA was reported as evidence for protective effects on anxiety in one study^57^. Several studies reported no effects of self-reported PA on schizophrenia^36,37,58^, bipolar disorder^36,37,48,54^, anxiety^54,59^ or PTSD^36^.

Overall, analyses of PA and cognitive traits were more consistent. Several studies reported no effects of accelerometer-measured PA on AD^60–63^. Wu et al.^64^ found that PA reduced AD risk in one dataset, but were not able to replicate these results in a validation set. One other study found a protective effect on AD^65^, but only when they used a lenient threshold to select instruments. Cheval et al.^66^ and Fang et al.^67^ reported that measured PA increased cognitive functioning, and reduced cognitive decline risk, respectively, while Li et al.^68^ reported that MVPA could have a negative impact on cognitive performance, and Liu et al.^63^ reported no causal effect on cognitive function. Liao et al.^60^, Baumeister et al.^61^, Zhang et al.^62^ and Wu et al.^64^ shared exposure/outcome summary statistics.

One study found that self-reported walking reduced the risk of AD^62^, while another study found that self-reported moderate PA, but not vigorous PA, increased the odds of developing AD^69^. No other study found effects of self-reported PA on AD liability and dementia^48,60,62,64,70^. One study reported that aerobic exercises, but no other kind of leisure PA, had an effect on cognitive function^71^. Finally, only one study investigated the effects of self-reported PA on intelligence and found no effects^72^. The quality of all the papers was assessed as low (−). Four studies fully shared exposure/outcome summary statistics^60–62,64^.

### Effects of device-measured and self-reported sedentary behaviours

There was evidence that sedentary time (accelerometer-measured) increased anxiety and decreased well-being^35^, and device-measured sedentary behaviours decreased anorexia and schizophrenia risk^36^. The quality of both papers was assessed as low (−). There was also evidence that self-reported TV watching, but not computer use and driving, increased depression risk^37,73^. The quality of these studies was moderate (±). Surprisingly, one study found that TV watching protected against bipolar disorder^37^. The quality of this study was moderate (±).

Negative effects of self-reported sedentary behaviours on depression (sedentary behaviour increased risk of MDD) were found^49,54^, albeit partially mediated by BMI^46^. After correction for BMI, Wang et al.^52^ found no effect of self-reported leisure sedentary time on depression. Chen et al.^74^ found positive causal effects of time spent watching TV and daytime napping on increased depression risk, while Li et al.^75^ found no effect of any sedentary behaviour phenotype on MDD risk. Dong et al. found associations between daytime napping and time spent playing computer games and bipolar disorder^54^. No effect of self-reported leisure sedentary (screen) time was found on MDD, ANX, SCZ or BP^39,54,59^.

While computer use reduced the risk of AD^73^, or had no effect on AD, dementia, or cognitive performance^76,77^, less intensive sedentary activities like TV watching were reported as detrimental for cognitive performance, function^78^, dementia, and AD Yang et al.^76^, Yuan et al.^77^, He et al.^73^ and Yuan et al.^77^ shared exposure/outcomes summary statistics. Zhang et al.^62^ found that neither self-reported nor measured sedentary behaviours were associated with AD. Finally, there was evidence of leisure sedentary time having a negative effect on intelligence, while sedentary behaviours at work had positive effects on intelligence. Sedentary commuting had no effect^72^.

### Effects of physical fitness phenotypes

The analyses of physical fitness traits on mental health showed more consistent results than the other PA phenotypes. Positive effects of low grip strength on depression were reported by Zhu et al.^79^. Higher grip strength, appendicular lean mass and walking pace had a protective causal effect on MDD^80–83^. Zhang et al.^80^, Wang et al.^81^ and Zhuo et al.^83^ shared exposure/outcomes summary statistics. Nanxi et al.^75^ found that left handgrip strength reduced MDD risk while right handgrip strength did not. Lv et al.^44^, however, found that the effect of walking pace and grip strength on depression did not remain significant after correction for BMI. They did find that lower appendicular lean mass (ALM) remained significantly associated with higher risk of depression. Finally, Vincent et al.^82^ found no clear evidence for an association between cardiorespiratory fitness and any depression outcome, and Lu et al.^84^ found no association between cardiorespiratory fitness and Alzheimer's risk. Most papers had a moderate quality rate (±), reflecting well-measured traits but capturing a highly pleiotropic phenotype.

Walking speed, ASM, ALM and handgrip strength were associated with cognitive function and performance^85–92^ although Liu et al.^92^ did not find a causal relationship between low hand grip strength and cognitive function. All these studies shared exposure/outcome summary statistics. Ye^93^ found that lower ALM and whole body lean mass values increased the risk of AD, whereas^93^ and Jones et al.^94^ found no effect of grip strength on AD, and Sun et al.^90^ found no effects on dementia.

A summarised version of all extracted data can be found in Table 1. Detailed records of all the extracted data can be found in Supplementary Table S4.

**Table 1:** Summary of the studies included and data extracted

Author	Year	Journal	Hypothesis	Result
(Choi et al.)	2019	JAMA psychiatry	Enhancing PA prevents MDD	Protective relationship between accelerometer-based activity and MDD. No significant relationship between self-reported activity and MDD.
(Wang et al.)	2022	Nature genetics	MVPA and LST are predictive of Alzheimer's, BIP or depression	Effects of lower LST on lower odds of depression. All effects mediated/confounded by BMI.
(Ye et al.)	2023	Aging cell	Sarcopenia-related traits have a causal effect on Alzheimer	Lower ALM is associated with increased risks of AD. Lower WBLM is associated with increased risk of AD.
(Cai et al.)	2022	European psychiatry	Individual behaviours like PA have a causal effect on 3 MH traits	PA has no effect on MDD, BIP or SCZ.
(Doherty et al.)	2018	Nature communications	PA has a causal effect on health outcomes	Small correlation between PA and risk for AD.
(Casanova et al.)	2023	BMC medicine	PA and sedentary time (ST) causally affect MH	Higher PA was associated with lower odds of depression and depression severity. Higher ST is associated with higher odds of anxiety.
(Papiol et al.)	2021	JAMA psychiatry	PA causally affects schizophrenia	No effect of self-reported or accelerometer-based PA on schizophrenia.
(Zhu et al.)	2023	Journal of cachexia, sarcopenia and muscle	Bidirectional causality between frailty and depression	Lower grip strength, slower walking pace and physical inactivity were all associated with a higher risk of depression.
(Choi et al.)	2020	The American journal of psychiatry	PA-related traits have a causal effect on depression	The deleterious effect of TV use on depression. Daytime napping linked to higher odds of depression.
(Iob et al.)	2023	Molecular psychiatry	PA and sedentary behaviour (SB) causally affect MH	Accelerometer-based average PA is associated with a lower risk of depression. Accelerometer-based SB decreased the risk of schizophrenia.
(Zhao et al.)	2023	Translational psychiatry	Bidirectional causality between MDD and PA	Causal relationships between strenuous sports, heavy do-it-yourself, other exercises and MDD. Physical inactivity caused an increased risk of MDD.
(Xu et al.)	2023	PloS one	Bidirectional causal relationships between different physical activities and intelligence	LST was negatively associated with intelligence, while SBW may have a positive effect on intelligence. MVPA and SC did not show significant effects on intelligence.
(Liao et al.)	2022	Frontiers in ageing neuroscience	Effect of PA on AD	No causal effects of PA on AD.
(Zheng et al.)	2023	PloS one	Effect of PA on MDD	Heavy DIY had an inverse association with MDD.
(Cheval et al.)	2023	Scientific reports	Bidirectional effects between PA and cognitive functioning	An increased fraction of both moderate and vigorous PA leads to increased cognitive functioning. No evidence of a causal effect of average physical activity on cognitive functioning.
(Zhang et al.)	2023	Medicine	Causal effect of sarcopenia-related traits on MDD	Grip strength and usual walking pace exhibited a direct causal effect on MDD.
(Wu et al.)	2021	Journal of sport and health science	Causal effect of PA on neurodegenerative disease	Effects of measured PA on AD.
(Wang et al.)	2023	World journal of psychiatry	Bidirectional effects between depression and sarcopenia	Inverse association of hand grip strength with depression.
(Ba et al.)	2024	Annals of general psychiatry	Bidirectional effects between leisure sedentary behaviour, PA and MH	Association between television watching and increased risk of depression. No evidence of a causal association between genetic liability for MVPA, accelerometer-assessed PA, computer use, or driving and the risk of depression. Driving has a slight effect in reducing the risk of schizophrenia, while leisure television viewing has a significant protective effect against the onset of bipolar disorder.
(Lv et al.)	2024	The American journal of geriatric psychiatry	Causal effects of sarcopenia-related traits on depression	Causal associations of ALM, walking pace and low hand grip strength with depression.
(Baumeister et al.)	2020	Neurology	Causal effect of PA on AD	No association between average accelerations and the risk of AD. The fraction of accelerations was unrelated to AD risk.
(Lu et al.)	2024	Scientific reports	Causal effect of sarcopenia-related traits on cognitive performance	Higher walking speed and ALM were causally and respectively associated with higher cognitive score, whereas a higher hand grip strength was positively associated with better cognitive performance.
(Chen et al.)	2024	Journal of affective disorders	Causal effects of lifestyle factors on MH	We found causal effects of strenuous sports or other exercise on anxiety disorders, bipolar disorder and MDD.
(Zhang et al.)	2022	Scientific reports	Causal effects of PA on AD	A protective relationship between walking and AD. Genetically predicted overall activity, sedentary behaviour and moderate-intensity activity were not associated with AD.
(He et al.)	2022	Genes	Bidirectional causal effects between sedentary behaviour and neuropsychiatric disorders	Consistent directional association between television watching and MDD, and a consistent relationship between computer use and a decrease in the risk of AD.
(Sun et al.)	2020	Journal of affective disorders	Causal effects of device-measured PA on BIP and SCZ	PA activity was associated with a lower risk of BIP. No significant relationship between PA and SCZ.
(Yang et al.)	2021	Frontiers in aging neuroscience	Causal effect of sedentary behaviours on AD and cognitive performance	Watching television was a detrimental factor in cognitive performance.
(Yu et al.)	2024	Frontiers in sports and active living	Effects of multiple physical activity patterns on psychiatric disorders	Light DIY activities showed no causal relationship with ADHD, depression, anxiety, or dementia. Heavy DIY activities were significantly negatively correlated with anxiety but had no causal relationship with ADHD, depression, or dementia. Vigorous exercise demonstrated no causal relationship with any of the studied psychiatric disorders. Aerobic exercise significantly reduced the risk of depression but showed no significant association with ADHD, anxiety, or dementia.
(Pasman et al.)	2024	medRxiv	Causal effects of many traits on MDD	Negative effect of exercise on MDD.
(Liu et al.)^114^	2024	Journal of affective disorders	Bidirectional effects between sedentary behaviours and delirium	Genetically predicted time spent watching television demonstrated a significant association with delirium.
(Nanxi Li et al.)	2022	General psychiatry	Causal effects of HGS (handgrip strength) on MDD	Left HGS is associated with a reduction in the risk of MDD.
(Yuan et al.)	2023	BMC public health	Causal effects of screen-based sedentary activity on AD and dementia	TV viewing was associated with a higher risk of AD.
(Jones et al.)	2021	Nature communications	Muscle weakness causally correlated with diseases	We observed no significant genetic correlation between HGS and Alzheimer’s disease.
(Chen et al.)	2020	medRxiv	Causal effects of daytime napping on several health outcomes	Daytime napping frequency was causally associated with increased risk for major depressive disorder and schizophrenia.
(Korologou-Linden et al.)	2022	Nature communications	Associations of PA and AD	Whole body fat-free decreased the risk of AD. Moderate PA but not self-reported vigorous activity increased the odds of developing AD.
(Zou et al.)	2026	Journal of Gerontological Nursing	Lower engagement in PA will have a negative effect on cognitive function	Frequent aerobic activities in the past 4 weeks have a positive genetic association with CF.
(Vincent et al.)	2026	Journal of Affective Disorders	Higher fitness and grip strength will be associated with a lower risk of depression and symptoms	Little evidence to support an effect of CRF on depression and its specific symptoms. Robust evidence linking higher weight-adjusted GS with lower odds of broad depression, as well as several specific symptoms.
(Zhang et al.)	2025	Frontiers in aging neuroscience	Association between sarcopenia and cognitive impairment	ALM and UWP had potential causal protective effects on cognitive impairment-related traits, while LHGS was only related to cognitive performance.
(Zhou et al.)	2025	Medicine	Association between sarcopenia and cognitive performance	Evidence supporting a causal role of SA-related traits in cognitive Function. Potential bidirectional causal relationship between walking pace and cognition.
(Du et al.)	2025	European Archives of Psychiatry and Clinical Neuroscience	Effect of physical activity on depression risk and major depressive disorder	Causal protective effects of physical activity on depression risk and major depression. Walking and heavy DIY are found to be causal, while the rest are not.
(Chen et al.)	2026	Journal of affective disorders	Effects of physical activity and its intensity on anxiety	MVPA exerted significant preventative effects on anxiety.
(Jiang et al.)	2026	Journal of affective disorders	Effects of physical activity and sedentary behaviour on MDD risk	Aerobic exercise (such as swimming, cycling and fitness training) was significantly negatively associated with a lower risk of MDD.On the other hand, the MR analysis of sedentary behaviour and sleep duration did not show clear causal links.
(Liu et al.)	2025	Mental Health and Wellbeing	Effects of physical activity on bipolar disorder	Heavy DIY activity was associated with a reduced risk of BD. No causal associations were found for the other four types of physical activity.
(Zhuo et al.)	2026	European Archives of Psychiatry and Clinical Neuroscience	Effect of sarcopenia on depression risk	ALM and left-handed grip strength had a bidirectional negative causal relationship with depression. Walking pace was negatively associated with depression risk; however, there was no inverse relationship. There was an inverse negative correlation between right-hand grip strength and depression. In the multivariate analyses, after adjusting for confounders, the associations of ALM and left-handed grip strength with depression were maintained.
(Sha et al.)	2025	JMIR Aging	Causal effects of sarcopenia on cognitive impairment	ALM, handgrip strength and gait speed all had a positive causal effect on the general cognitive function score.
(Galimberti et al.)	2025	Nature Genetics	Many outcomes, but an exploratory paper	No effect of all-leisure PA on Alzheimer's.
(Chen et al.)	2025	Journal of affective disorders	Effect of grip strength on cognitive function	Results indicated a bidirectional association between grip strength and cognitive impairment in people over sixty years of age.
(Sun et al.)	2025	Am J Transl Res	Effects of hand grip strength on cognition	Positive causal effects of hand grip strength on cognitive function. No directional causal effects of cognition function on hand grip strength.
(Xu et al.)	2025	Journal of affective disorders	Effects of physical activity on depression onset	Increased physical activity was associated with a reduced risk of depression. Specific activities contributing to this association included light DIY tasks, recreational walking, other exercises, heavy DIY tasks and strenuous sports (e.g. running, competitive sports).
(van Hooijdonk et al.)	2025	Psychological Medicine	Bidirectional effects between physical activity and depression/anxiety	No significant effect of physical inactivity on the risk of depression or anxiety. No significant effect of depression or anxiety on physical inactivity.
(Dong et al.)	2025	Journal of affective disorders	Effects of multiple lifestyles on multiple psychiatric outcomes	Leisure screen time and napping were significantly associated with MDD. No association with ANX. Playing computer games and napping were significantly associated with BIP. No effects of physical activity on anything.
(Duan et al.)	2024	Geriatrics Gerontology	Effects of sarcopenia on cognitive performance	Bidirectional effects between ALM, HGS, walking pace and cognitive performance.
(Lu et al.)	2024	EPMA	Effects of cardiorespiratory fitness on Alzheimer's	No association between cardiorespiratory fitness and Alzheimer.
(Lyu et al.)^115^	2024	Brain Sciences	Effects of PA, SB and BMR on risk of PTSD, MDD	For PTSD, the protective effect of physical activity, but the wide confidence interval introduced significant uncertainty. In the case of MDD, none of the MR methods showed a significant association with physical activity. Similar results for SB and BMR.
(Bai et al.)	2024	Frontiers in Psychology	Effect of different lifestyles on cognitive function	Unidirectional causal effect of TV watching on cognitive function.
(Li et al.)	2024	Frontiers in aging neuroscience	Effects of MPVA on cognitive performance	MVPA had a significant effect on cognitive performance.
(Fang et al.)	2024	Frontiers in Psychology	Bidirectional effects of MVPA on cognitive performance	Bidirectional effects between MVPA cognitive performance.
(Lu et al.)	2025	Journal of affective disorders	Effects of individual behaviours and anxiety	No effects of any kind of PA, daytime napping, computer or driving on anxiety. Effects of TV watching on anxiety.
(Nan-Xi et al.)	2024	Psychiatric Genetics	Effects of SB on MDD.	No association was observed for SB on MDD.
(Liu et al.)	2024	PloS One	Effect of sarcopenia traits on cognitive impairment	ALM had a causal relationship with walking pace on cognitive function. According to Bonferroni correction analyses, no causal relationship was identified between low grip strength and cognitive function. Similar results were found in the other direction of effect.
(Liu et al.)	2024	Frontiers in aging neuroscience	Effects of physical activity on cognitive function	The MR results indicated no causal link of PA (AVPA, MVPA and VPA) on CF, Alzheimer’s disease and dementia.

Including, from left to right (Author, Year, Journal, Hypothesis and Result).

*MDD* Major Depressive Disorder, *AD* Alzheimer’s Disease, *PA* Physical Activity, *MVPA* Moderate to Vigorous Physical Activity, *LST* Leisure Sedentary Time, *SCZ* Schizophrenia, *BIP* Bipolar, *ALM* Appendicular Lean Mass, *WBLM* whole-body lean mass, *SC* Sedentary commuting, *SBW* Sedentary behaviour at work.

## Discussion

This review synthesised evidence from 61 Mendelian Randomisation (MR) studies examining the potential causal effects of PA, sedentary behaviour and physical fitness on brain health outcomes. Overall, the findings do not offer consistent support for a broadly protective role of PA across outcomes. Rather, the evidence suggests that different types of PA (i.e. being active, sedentary, or fit) show distinct patterns of association with brain health.

PA measured as being physically active (e.g. self-reported PA) did not show consistent evidence for protective effects on depression, schizophrenia, anxiety, or bipolar disorder. This contrasts with observational research, which more consistently links PA to reduced risk of these mental health conditions as well as improved cognitive function^22,41,43^. This discrepancy may be due to confounding and reverse causation in observational studies, where individuals with better mental health may be more likely to engage in PA. Additionally, self-report PA measures are prone to misclassification and recall bias, potentially diluting true causal effects in MR analyses^42,95^. Out of several activity types and intensities, aerobic exercises, compared to strenuous sports, do-it-yourself activities and light-intensity PA, showed the most consistent positive results on depression risk reduction. These considerations emphasise the importance of using diverse evidence across methods when evaluating the mental health benefits of being physically active.

In a similar fashion, MR studies examining sedentary behaviours found mixed evidence for effects on brain health. Time spent watching TV was most consistently associated with increased depression risk. However, the causal effects did not survive BMI correction in several studies. These findings partially align with observational research showing that prolonged sedentary time is associated with poor mental health and cognitive outcomes, but also with worse general health^96–98^. However, caution is still warranted. Sedentary behaviour is closely (genetically) linked to socioeconomic status, physical illness and other potential confounders that may also influence genetic liability to mental health problems. In MR analyses, this association may further be biased by mechanisms like assortative mating, population stratification and dynastic effects^99^. Such biases are particularly relevant for lifestyle-related phenotypes like sedentary behaviour, which may be shaped by family environments and social context. To strengthen causal inference in this context, family-based MR designs, which compare siblings or use parental genotypes to control for shared environmental and genetic background, would be especially valuable.

Physical fitness traits such as hand-grip strength and appendicular lean mass showed the most consistent evidence for beneficial effects, both on mental and cognitive health. Although these measures are not strictly PA phenotypes, they can serve as a proxy, are objectively easier to measure than exercise (e.g. amount of muscle mass versus frequency of intense sporting), and are also representative of the fitness of individuals that are unable to or do not engage in intense PA like running or swimming^100^. Our findings align with recent observational studies^101,102^, though many such studies have focused on older adults^103,104^. This highlights the need, in future research, to consider age-related comorbidities—such as osteoporosis, arthritis, or cardiovascular disease—which may contribute to both decreasing brain health and physical fitness. Therefore, these findings need to be interpreted cautiously, as fitness partially captures frailty and ageing, which in turn may also affect health and cognition.

The overall quality of the included MR studies was moderate to low. Of the 61 studies, 28 were rated as ‘sufficient’ and 32 as ‘low’, with only one rated as ‘good’. Although only one of 46 studies reporting mean F-statistic showed below-standard (*F* < 10) metrics, and GWASs sample sizes averaged above 100,000, several studies used lenient SNP-selection thresholds, included few genetic variants, or relied on coarse PA phenotypes, any of which may compromise instrument quality in ways not reflected by standard metrics. We are therefore unable to fully rule out instrument-related sources of bias, underscoring the need for cautious interpretation of the findings. Especially in light of limitations such as weak instrument strength, potential for horizontal pleiotropy and inconsistent phenotype definitions across studies. Additionally, multiple studies repeated identical analyses using the same exposure-outcome pairs and the same underlying GWAS data. Rather than providing independent replication, this redundancy risks overstating the consistency of the evidence and limits the incremental value of subsequent studies, complicating efforts to distinguish genuinely novel findings from reiterations of existing results.

These caveats may partially explain the discrepancy between intervention studies finding more consistent effects of PA on brain health^105^ and MR studies, which seem to be less consistent and generally do not find strong effects. Of special interest are two key differences. First, RCTs are targeted studies that measure the direct, short-term impact of an intervention, while MR studies estimate the lifelong influence of genetically predicted exposures. This means that the intervention effects may not be representative of a daily exposure and that the effects might be different due to interventions being short-term and MR representing longer-term effects. Second, RCTs try to minimise confounding by design while MR relies on strong assumptions about the validity of genetic instruments and confounding^33^, which may be difficult to meet for complex traits like PA^106^. For example, for traits like alcohol use, there are clear biological mechanisms through which a SNP in an alcohol metabolising gene affects alcohol use behaviour, but for behaviours like TV watching, the mechanism is not clear. A GWAS could capture genetic variants related to traits other than the exposure (i.e. SES), which may affect the target phenotype. This would result in horizontal pleiotropy, violating the exclusion restriction assumption and leading to biased estimates. Such differences highlight the importance of using strict sensitivity methods and integrating evidence across different study designs.

One way to conduct more reliable causal inference is through a process of ‘evidence triangulation’. By combining different methods and data sources, triangulation helps increase the credibility and validity of the results by checking if different approaches lead to the same conclusion^107^. This is important for all MR studies, but especially for those with traits that are highly polygenic and complex (like PA), and where the path between SNP, exposure and outcome is not completely understood^108^. Notably, several of the most recent studies included in this review adopted a triangulation strategy, several of them supporting the results of this review across different methods^53,55,83,88,89^. This pattern already underscores a shift in the field toward more integrative and methodologically rigorous approaches to causal inference.

Differences between self-reported and device-measured PA likely contribute to the variability in findings that we report. Self-report measures are prone to recall bias and overreporting, though they can capture a broader range of behaviours over longer periods. Device-based measures are more precise but focus primarily on locomotor activity, potentially missing non-aerobic or resistance-based exercise^42,95^. A genetic correlation of 0.49 has been reported between self-reported and device-measured PA GWASs^30^, indicating partial but incomplete overlap. In our review, protective effects on depression were more often found in MR studies using device-measured PA. However, it is unclear whether the device-measured signal reflects a genuinely stronger causal effect or simply different statistical properties (e.g. smaller sample sizes and differences in instrument strength) compared to self-reported PA. It has also been suggested that current mood and cognitive biases may influence self-reported activity more than objective measures^109^. In future research, studies should use both types of measures and apply stringent sensitivity analyses to improve the reliability of genetic instruments.

This study is not exempt from limitations. First, we focused on physical activity, inactivity and fitness as exposures, with multiple phenotypes per exposure, resulting in a large number of exposure phenotypes in a limited number of studies. While this reflects the complex and multidimensional nature of PA and related constructs, it also introduces heterogeneity in how these constructs are operationalised. In addition, the level of granularity of these exposures is limited by the underlying GWASs, which often capture broad and composite behavioural measures rather than detailed characteristics such as intensity, frequency, duration, or contextual domains. As a result, it remains difficult to disentangle dose–response relationships or context-specific effects within a Mendelian Randomisation framework. Future research would benefit from more refined and harmonised GWAS phenotypes that better capture the multidimensional nature of PA behaviours. Second, most studies relied on GWASs from similar samples, particularly the UK Biobank, which limits generalizability. UK Biobank participants are predominantly of European ancestry, older, wealthier, healthier, and more educated than the general population, with lower rates of smoking, obesity, and chronic disease^110^. Critically, this sample overlap also undermines the independence of findings across studies: of the 61 included studies, only 26 (43%) used independent summary statistics for a given exposure–outcome pair. As a result, instead of having multiple studies showing non-overlapping evidence, we see a smaller number of underlying genetic instruments analysed repeatedly across studies. This non-independence likely inflates the apparent consistency of both null and positive findings and should be considered when weighing the overall strength of evidence. Third, title and abstract screening and initial data extraction were completed by a single reviewer, which may increase the risk of selection bias or information omission. However, inclusion criteria were defined a priori by three reviewers, a random subset of records was double-screened to confirm high agreement between reviewers, MR scoring (which involved full-text screening) was performed by all three reviewers, and all full-text assessments and uncertain cases were discussed among three reviewers, likely reducing the risk of bias. Lastly, given the genetic epidemiology and biomedical nature of MR studies, the literature search was performed on PubMed, which provides extensive coverage of genetic and epidemiological studies and is the primary database in which MR studies are indexed and medRxiv, and not on other more specific databases under the conviction that the incremental yield of eligible studies from these databases would be highly limited.

In conclusion, this review of MR studies supports a moderate protective role of physical fitness traits on mental health, alongside a stronger protective effect on cognitive outcomes, as well as slight negative effects of sedentary behaviours on depression. In contrast, no clear support was found for causal effects of general physical activity on mental health outcomes, regardless of whether activity was self-reported or device-measured. Importantly, the apparent lack of association between the genetic components of physical activity and brain health does not preclude a substantial role for environmental influences. In addition, the effects of fitness go through physical pathways as well as potentially through ageing and frailty effects. These findings highlight that using MR as a standalone tool for causal inference is far from optimal for complex traits where there is no clear biological pathway between the included genetic variants and the exposure of interest. Despite the numerous advantages of MR, the difficulty in obtaining reliable biologically-informed instruments is one of the current challenges in the field. We therefore urge researchers to combine MR with observational, interventional and family-based designs, while prioritising consistent phenotype definitions and robust sensitivity analyses to strengthen causal inference.

## Methods

### Literature search and review

This study was pre-registered at PROSPERO (CRD42024514302; https://www.crd.york.ac.uk/prospero/display_record.php?RecordID=514302); registration date 22 June 2024. Available literature was systematically reviewed by searching PubMed and preprint server medRxiv for all MR studies including any PA measure (e.g. exercise, sports, training) as an exposure for causal effects on a range of psychiatric, mental well-being and neurocognitive outcomes (‘brain health’). No date restriction was applied. We restricted our search to English-language publications (search strategy provided in Supplementary Information 1). The search was conducted on 2nd of April 2026. No ethical approval was required for this study.

Search output was downloaded and uploaded to Rayyan^111^, an online tool to organise and simplify literature reviews. Duplicates were identified and excluded. Next, potentially eligible studies were selected to be included based on title and abstract. We followed PRISMA guidelines in extracting and selecting the data. To ensure consistency in applying the inclusion and exclusion criteria defined a priori by three researchers (A.T., M.W. and J.T.), a random subset of 100 records was independently screened by two reviewers (A.T. and M.W.). The selection results were compared, and a 97% alignment was achieved (Cohen’s kappa = 0.78). Cases of disagreement were reviewed individually and discussed by three reviewers (A.T., M.W. and J.T.), and were ascribed to differences in phenotype inclusion. One of the reviewers was more lenient on the physical fitness phenotypes, but all reviewers settled for a stricter revision. Given this high alignment, the remaining records were screened by a single reviewer (A.T.). Full-text articles were reviewed independently, and any uncertainties regarding eligibility were discussed among all three researchers to reach a final decision.

### Inclusion and exclusion criteria

According to PICO(S) guidelines, we report our population of interest to be human participants of any age or health status (P). Our interventions (I) were individual differences in PA, sedentary behaviour, and related constructs defined broadly, and encompassing objective measures (grip strength tests, accelerometer data, etc.) and self- or proxy-reported measures (questionnaires, journals, etc.). Since the (in)ability to engage in PA is a major determinant of PA-related traits, we additionally included physical fitness traits (muscle mass, strength), which are often used as proxies for PA among older populations. Measures not exclusively linked to PA, for instance, the frailty index (which incorporates several health parameters beyond PA), were excluded.

For comparators (C), MR studies inherently estimate the effect of higher versus lower genetic liability for the exposure. By synthesising these estimates, we aimed to evaluate causal relationships between PA-related behaviours and brain health outcomes.

For the outcomes (O), all (symptoms of) mental health disorders diagnosed by a professional or measured through self-report on (standardised) questionnaires were included, as well as any cognition-related outcome. Within neurodegenerative outcomes, we differentiated between those primarily associated with cognition (e.g. Alzheimer’s disease (AD), dementia) and those with a predominantly physical component (e.g. ALS, Parkinson’s disease). The former were included, whereas the latter were excluded.

Original research studies (S) employing MR analyses to explore the causal effect of any PA exposure(s) on the outcome(s) of interest were included. Papers where PA constructs were only treated as an outcome were excluded. Review papers were excluded.

### Mendelian Randomisation

MR is an instrumental variable approach that employs (a set of) genetic variants (most commonly single-nucleotide polymorphisms, SNPs) to proxy an exposure and test its effects on an outcome.

Somewhat similar to an RCT, the random segregation of alleles (genes) inherently splits the population into (genetically) ‘exposed’ and ‘controlled’ groups. Because segregation is random, it is unlikely that being a part of one or the other group is influenced by confounding effects that bias conventional epidemiological analyses.

**Fig. 3:**
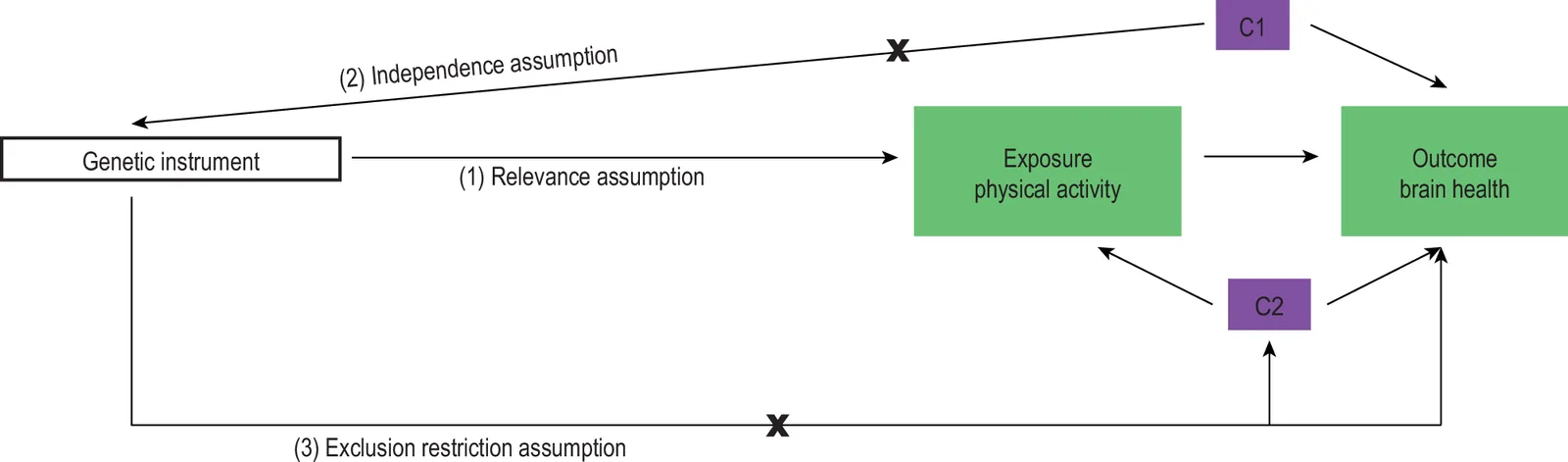
Visual representation of the 3 core assumptions of Mendelian Randomisation (MR). The genetic instruments used serve as proxies of the exposure—in this example, where physical activity is represented as exposure, the genetic instruments proxy physical activity. C1 refers to confounding mechanisms such as assortative mating and population structure that affect both allele distributions in the population and the outcome, and C2 refers to confounders that affect the exposure and the outcome. Figure created with Adobe Illustrator.

MR relies on 3 core assumptions (Fig. 3): (1) genetic variants used as instrumental variables must be associated with the exposure of interest (relevance); (2) genetic variants used as instrumental variables should be independent from confounding factors (e.g. assortative mating, population structure) also affecting the outcome (independence), and (3) genetic variants do not influence the outcome in any other way than through the exposure (exclusion restriction). Horizontal pleiotropy is when a genetic variant influences the outcome independently of the hypothesised exposure, and it can undermine the assumptions. Various MR sensitivity analyses have been developed to assess potential deviations from these assumptions.

‘One-sample MR’ uses individual-level data on the exposure and outcome from the same study sample. ‘Two-sample MR’ derives these associations from two independent GWAS samples instead, allowing large (publicly available) GWAS datasets to be conveniently combined.

### Scoring MR papers

We independently evaluated the quality of the MR papers based on a scoring system developed by Treur et al.^112^ (Supplementary Information 2), which was cross-checked with the most recent MR guidelines^113^. This system allows for a systematic and rigorous risk-of-bias assessment of multiple aspects of the studies, rather than a reporting checklist.

The scoring system is based on several key features: (1) phenotype measurement (including sample size and quality of the exposure and outcome measurements), (2) instrument strength (*p*-value threshold used to select SNPs, number of SNPs, biological knowledge of genetic variants, F-statistic for instrument strength, percentage of variance that the instrument explains) and (3) sensitivity methods used. Each study was given a final score of ‘–’ (insufficient), ‘±’ (sufficient) or ‘+’ (good). If both the sample size and the key analytical method requirements were met, the study score was considered sufficient (±). When a study had a good sample size and analytical methods and additionally used extensive (sensitivity) methods, a good score (+) was given. We applied a reasonable doubt principle, where we inferred parameters that were not reported but that can be known based on other literature (e.g. if a sample size was not reported but we knew it from other studies or prior research). If the required parameter was missing and could not be inferred, we report a ‘?’. The selected manuscripts were divided into two sets. Two of three researchers (A.T., M.W., J.T.) independently scored each set and later compared the scores. In case of disagreement, all three researchers discussed the differences and reached a final decision.

### Strategy for data synthesis

Our data synthesis strategy was to outline the results, strengths and weaknesses of the included MR studies examining the relation between PA, fitness, sedentary behaviours and brain health. We extracted information on the studies’ hypotheses, measure(s) of PA and brain health used and results of the main MR analysis (one reviewer AT). Additionally, we assessed differences in findings between studies that used objectively measured versus self-reported PA (Supplementary Information 3). MR exposures were first extracted and reported according to the original GWAS phenotypes used in each MR study. Given that MR estimates are inherently tied to the specific genetic instruments derived from these GWASs, phenotypes were not initially harmonised or merged at the instrument level. However, to facilitate interpretability, studies were grouped by exposure type (physical activity, sedentary behaviours and physical fitness phenotypes) and by measurement method (objectively measured vs. self-reported). Outcomes were similarly grouped into mental health traits and cognitive traits, including neurodegenerative disorders primarily associated with cognition. First data extraction started on 01/08/2024.

## Supplementary information

Supplementary information

Supplementary information

Supplementary Data

## Data Availability

Tables summarising the data extracted are available in the Supplementary Data tables. GWAS summary data are available in the original study papers. No new data were created or analysed in this study. No custom code was created in this study.
